# Magnitude and predictors of common mental disorder among people with HIV/AIDS in Ethiopia: a systematic review and meta-analysis

**DOI:** 10.1186/s12889-020-08800-8

**Published:** 2020-07-07

**Authors:** Zelalem Belayneh, Birhanie Mekuriaw, Tsegaye Mehare, Seid Shumye, Mekonnen Tsehay

**Affiliations:** 1 Department of Psychiatry, College of Health and Medical Science, Dilla University, Dilla, Ethiopia; 2 Bio-Medical Department, College of Health and Medical Science, Dilla University, Dilla, Ethiopia; 3 Department of Psychiatry, College of Medicine and Health Science, Wollo University, Dessie, Ethiopia

**Keywords:** Common mental disorder, Mental distress, Psychological distress, Distress, HIV/AIDS, Mental illness, HAART, ART, Ethiopia

## Abstract

**Background:**

Common mental disorders are frequent psychiatric comorbid conditions among people with HIV/AIDS. The presence of such psychiatric disorders negatively affects the treatment adherence, viral load suppression, quality of life, treatment outcomes and functionality of people with HIV/AIDS. However, available studies in Ethiopia have a great variation and inconsistency of reported results have been observed regarding the magnitude and associated factors of common mental disorder. Thus, conducting a systematic review and meta-analysis of existing literatures can have a paramount importance to show its summarized figure.

**Methods:**

Literatures search was performed using databases (PubMed/Medline, Science Direct and PsycINFO. Grey literatures were also searched from Google and Google Scholar. Data were extracted from primary studies using a data extraction format prepared in Microsoft Excel and exported to STATA-version 14 statistical software for analysis. The I^2^ test was used to assess the heterogeneity of primary articles. The result of the test showed that there was heterogeneity between primary studies. This leads us to execute a random effect meta-analysis to estimate the pooled prevalence of common mental disorder with corresponding 95% confidence interval.

**Results:**

A total of 13 primary studies comply with the inclusion criteria were included in this systematic review. The pooled prevalence of common mental disorder was found to be 28.83% (95% CI: 17.93, 39.73) among people with HIV/AIDS in Ethiopia. The highest prevalence of common mental disorder (35.20%) was observed among studies in which Kessler-10 was used as a screening tool. Single marital status (OR = 1.83; 95%CI: 1.03, 3.27), HIV/AIDS-related stigma (OR = 2.21; 95%CI: 1.68, 2.90) and current job unavailability (OR = 1.38; 95%CI: 1.01, 1.88) had statistically significant association with common mental disorder.

**Conclusion:**

The result of this review showed that nearly one among three individuals with HIV/AIDS is suffering from common mental disorder in Ethiopia. This calls a need to integrate the mental health and psycho-social support into the HIV/AIDS care.

**Trial registration:**

PROSPERO- CRD42019132402. Registered on 05/08/2019.

## Background

Common mental disorder (CMD), sometimes called mental distress or psychological distress is a gross name given to describe a range of psychological disturbances explained by the presentation of depressive, anxiety, and/or somatic symptoms [1, 2]. Such groups of psychological disturbances often indicated by repeated visits to primary health care practitioners without the resolution of the problem [3–5].

Nowadays, CMD becomes a common public health problem both in developing and developed populations [1, 6, 7]. Its global prevalence is 17.6% [8] in general population. The WHO estimates the magnitude of common mental disorder to be 8% among African regions [9].

CMD becomes more common among people with chronic medical conditions, particularly HIV/AIDS [10, 11]. For example, a study conducted in southwest Ethiopia revealed that 75% of people with HIV/AIDS are suffering from CMD. The chronicity of AIDS after the innovation of Highly Active Antiretroviral Therapy (HAART) [12], HIV/AIDS-related stigma, functional impairment, the direct brain effect of the virus, medication side effects, other opportunistic infections and comorbid conditions are some of the contributing factors for the higher burden of CMD among people with HIV/AIDS [13, 14].

The presence of comorbid CMD can have negative and complicated impacts on the treatment outcome and quality of life of individuals. For instance, CMD diminishes the treatment adherence [15], viral load suppression, immunity [16] and functionality [17] of people with HIV/AIDS. People with CMD often become hopelessness and have lack of assertiveness to perform protected sex which may together accelerate the risk of further HIV transmission. Moreover, people often initiate abuse of psycho-active substance like Khat (a green plant having stimulant effect when its leaves are being chewed), alcohol, cigarette [18, 19] as a self-medication to get relief from symptoms of common mental disorder [20, 21].

Although there are some studies reporting the prevalence and predictors of CMD among people with HIV/AIDS in Ethiopia, a great variation and inconsistency of reported results have been observed [22, 23]. Therefore, the aim of this systematic review and meta-analysis was to estimate the pooled prevalence of common mental disorder among people with HIV/AIDS in Ethiopia. This systematic review and meta-analysis will help for policymakers, health personnel, non-governmental organizations and other concerned bodies to design appropriate preventive and interventional programs for people with HIV/AIDS. The result of this study will also be used as baseline information for further research works.

### Objectives

The first objective of this review was to measure the pooled prevalence of common mental disorder among people attending anti-retroviral therapy (ART) clinics of Ethiopia. This can help for policymakers and clinical practitioners to integrate the mental health and psycho-social support service as an added treatment modality to the HIV/AIDS care. The second objective was to compare the pooled prevalence of common mental disorder among studies based on the screening tools of CMD. Finally, this systematic review and meta-analysis identified predictors of common mental disorder among people with HIV/AIDS. This can be helpful for the prevention, early identification and interventions of common mental disorder among people with HIV/AIDS by preventing and/or minimizing its risk factors.

## Main text

### Methods

#### Searching methods and study selection

Systematic search of both published and unpublished primary articles were conducted using different databases (PubMed/MEDLINE, Science Direct and PsycINFO). Grey literatures were identified from Google and Google scholar. We also performed literatures search using direct web sites of local (Ethiopian) journals. Articles related to the prevalence and/or associated factors of common mental disorder among people living with HIV/AIDS were retrieved. Key terms used to retrieve primary articles were (((((((Common mental disorder) OR Psychological distress) OR Mental distress) AND HIV/AIDS) OR Anti-Retroviral Therapy) OR Highly Active Anti-Retroviral Therapy) AND Ethiopian).

All relevant articles available online until May 15^th^, 2019 were considered for this systematic review and meta-analysis. This systematic review and meta-analysis was carried out in accordance with the Preferred Reporting Items for Systematic reviews and Meta-Analyses (PRISMA) guideline [24] (Additional file 1). The review protocol has been registered in the International Prospective Register of Systematic Reviews (PROSPERO) with a registration number of “CRD42019132402”.

All searched articles were evaluated for their eligibility to be included in the review. First, studies were evaluated by reading their titles/abstracts. Papers considered as relevant after reading their titles and abstracts were selected for further evaluation by reading their full-text. After the full text evaluation, thirteen papers were found to be eligible and included in this systematic review and meta-analysis.

### Eligibility criteria

All retrieved primary studies were reviewed and checked for their eligibility to be included in the systematic review and meta-analysis based on the criteria listed below.

### Inclusion criteria

#### Study area

Research articles conducted across people with HIV/AIDS in Ethiopia were considered as eligible.

#### Study design

Observational studies (cross-sectional, case-control and cohort studies) with original data reporting the prevalence of common mental disorder and/or its associated factors were considered.

#### Language

Literatures written in English language were included.

#### Population

Studies conducted among adults (age greater than or equal to 18 years) attending ART clinics in Ethiopia were included.

#### Publication issue

Both published and unpublished articles available online until May 15^th^, 2019 were included.

### Exclusion criteria

In the full-text evaluation of articles, we considered study settings, target populations, age of respondents, study design, paper quality and outcomes of interest. Primary studies conducted among minors (age less than 18 years old), papers of non-Ethiopian population or non-HIV/AIDS patients, non-observational studies, papers with low quality score and studies that did not have reports of magnitude, and/or associated factors of CMD were excluded.

### Data extraction

Three authors (ZB, BM and TM), independently extracted all necessary data using a data extraction format prepared in Microsoft Excel. For the first objective, the data extraction format had sections of name of first author, publication year, region of the country where studies are conducted, screening tools of CMD, sample size, response rate, and prevalence of common mental disorder. Each section stands with a single column and rows were filled with data from each primary study. For the second objective (factors associated with common mental disorder), a data extraction format prepared in a two by two table form was used (Additional file 2). Any disagreements during data extraction were identified and resolved through discussion.

### Outcome measurements

This systematic review and meta-analysis had three main outcomes; the pooled prevalence of CMD, the variation of CMD based on screening tools and predictors of CMD among people with HIV/AIDS. The prevalence of common mental disorder was calculated by dividing the number of participants screened positive for common mental disorder to the total number of samples and multiplied by hundred. Regarding factors associated with common mental disorder, odds ratio was calculated from primary studies using two by two tables. Finally, we performed a sub-group analysis based on screening tools used to measure CMD.

### Quality assessment

The quality of primary studies included in this systematic review and meta-analysis were assessed using the Newcastle-Ottawa Scale adapted for cross-sectional study quality assessment [25]. The tool has different indicators consisting of three main parts; the first part has five components used to assess the methodological quality of each study; the second section examines the comparability of primary studies, and the last part measures the quality of original articles with respect to their statistical analysis. Three authors (ZB, BM, and TM), evaluated the quality of each original article independently using this assessment tool. Inconsistencies between assessors were solved through discussion and the average score of different assessment results were used. Articles fulfilling at least 50% of the quality assessment criteria score were included in this review.

### Statistical procedures

The extracted data were imported from the Micro Soft Excel data extraction format to the STATA (Version-14.0 software) for analysis. The standard error of prevalence was calculated using the binomial distribution formula for each original article. The heterogeneity of primary studies was checked using χ^2^ test and I^2^ test [26]. In this review, there was significant heterogeneity among primary studies as explained by I^2^ = 99.20% and *P =* 0.000. Therefore, a random-effects meta-analysis model was executed to estimate the Der Simonian and Laird’s pooled effect of common mental disorder and its associated factors. Publication bias was also examined among primary studies using Egger’s correlation tests and funnel plot [27, 28]. The result of these tests showed that there was no small study effect among primary studies as evidenced by *P* = 0.742 for the Egger’s test, and there was a relatively symmetrical distribution of a funnel plot. Furthermore, a subgroup analysis was performed among primary studies based on screening tools used to measure CMD to minimize the random variations between their point estimates.

## Results

### Search results

**Fig. 1:**
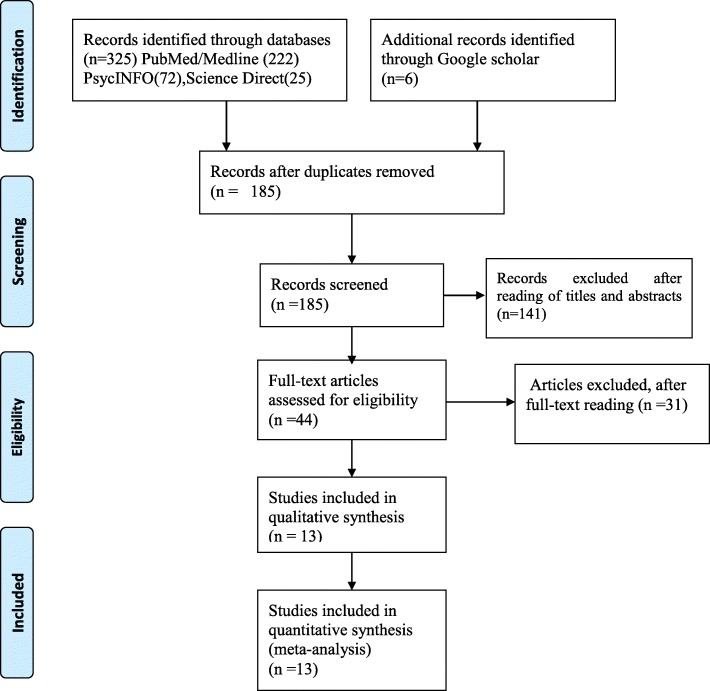
Flow chart explaining the selection of primary studies

In the first step of our literature search, a total of 325 articles were retrieved using databases (PubMed/Medline = 222, PsycINFO = 72, Science Direct = 25). Additional six articles were also searched from Google and Google scholar. First, all searched articles were imported to End-note, and duplications were removed. After the removal of duplications, there were 185 articles considered eligibility for title and abstract evaluation. Accordingly, 141 articles were excluded and remaining 44 articles were considered for further full-text evaluation. After the full-text reading, 31 articles were further excluded due to differences in the study population, study design and outcome of interests. Finally, thirteen papers were found as eligible to be included in this systematic review and meta-analysis (Fig. 1).

### Original articles characteristics

After a thorough assessment of papers, thirteen primary studies were identified as eligible to be included in this systematic review and meta-analysis. These studies were conducted across five different regions of the country (three at Amhara, six at Oromia, two from Southern Nation, Nationality and People SNNPR, one from Addis Ababa, and the other one from Harare (eastern Ethiopia). However, a highly variable and inconsistent results of CMD ranging from 7.80 to 75% was reported. Regarding assessment tools of CMD, seven studies used the Kessler 10- item questionnaire, two used the Hospital Anxiety and Depression Scale (HADS) and other four used the Self-Reported Questionnaire (SRQ-20) (Table 1).

**Table 1:** Summary of 13 primary studies of people with HIV/AIDS in Ethiopian included in the systematic review and meta-analysis, 2019

Region	Author	Publication year	Assessment Tool	Sample size	Response rate (%)	Quality score	Prevalence (95% CI)
Amhara (3)	**Selamawit** Z et al. [29]	2015	SRQ-20	412	97.6	9	24.3 (20.16, 28.44)
	Elizabeth M et al. [30]	2014	K-10	349	100	9	14.90 (11.16, 18.64)
	Elyas A et al. [22]	2019	SRQ-20	422	100	8	7.80 (5.24, 10.36)
Oromia(6)	Amare D et al. [31]	2013	K-10	455	100	6	21.80 (18.01, 25.59)
	Amare D et al. [32]	2010	K-10	465	100	8	46.70 (42.17, 51.23)
	Matiwos S et al. [33]	2014	K-10	389	97	7	45.20 (40.25, 50.15)
	Matiwos S et al. [34]	2015	K-10	389	97	7	13.36 (9.98, 16.74)
	Angela M. et al. [35]	2018	K-10	1175	99.5	9	29.50 (26.89, 32.11,)
	Angela M. et al. [23]	2018	K-10	722	100	8	75.00 (71.84, 78.18)
Addis Ababa(1)	Getachew T et al. [36]	2016	HADS	417	100	8	24.50 (20.37, 28.63)
SNNPR(2)	Solomon H. &Girma T [37].	2014	HADS	500	100	9	11.20 (8.40,13.95)
	Bereket D et al. [38]	2019	SRQ-20	294	98.7	9	32.70 (27.34,38.06)
Harari (1)	Aboma M et al. [39]	2019	SRQ-20	420	100	9	28.10 (23.08, 32.40)

*Abbreviations*: *AIDS* Acquired Immune Deficiency Syndrome, *HAD* Hospital Anxiety and Depression Scale, *HIV* Humane Immune Virus, *K-10* Kessler-10, *SRQ* Self-Reporting Questionnaire

### Pooled prevalence of common mental disorder

**Fig. 2:**
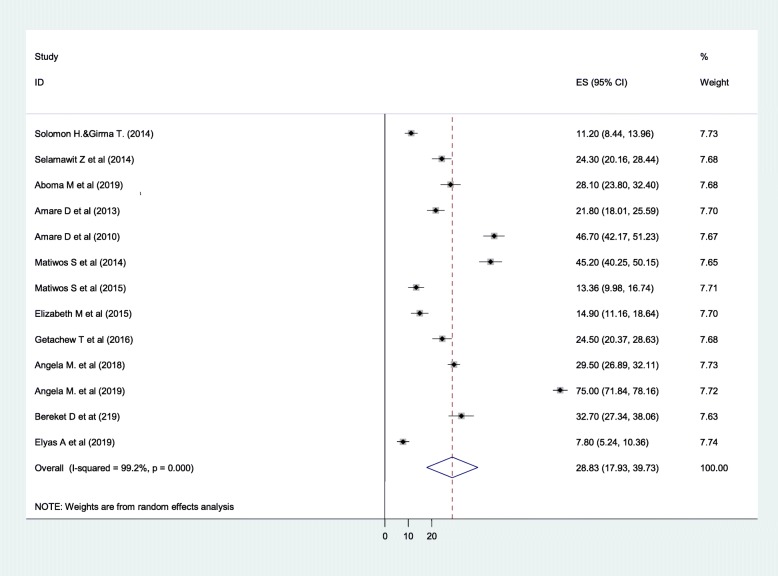
Forest plot for the pooled prevalence of common mental disorder

The pooled prevalence of common mental disorder was calculated from 13 primary studies fulfilling the inclusion criteria. There were a total of 6409 respondents attending ART clinics in Ethiopia across all the 13 primary studies. Accordingly, the pooled prevalence of common mental disorder among people with HIV/AIDS was found to be 28.83% (95% CI: 17.93, 39.73) (Fig. 2).

### Subgroup analysis

**Fig. 3:**
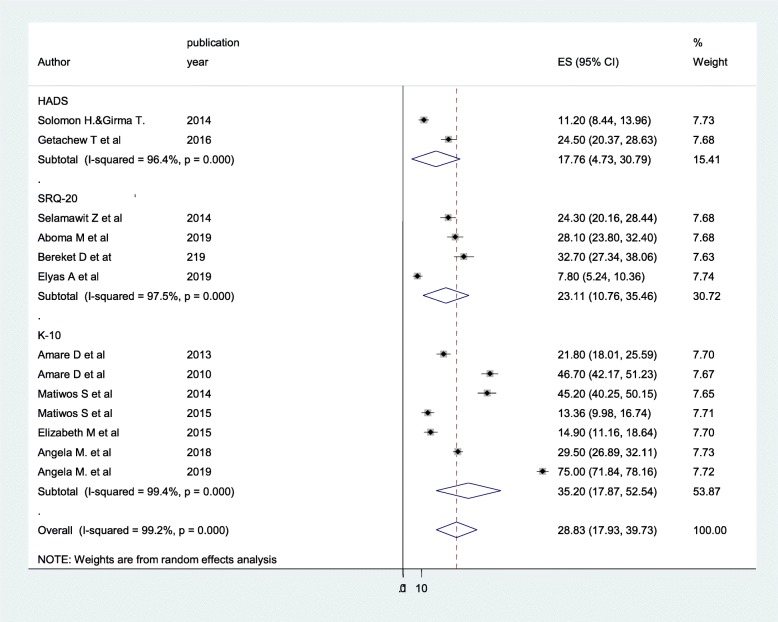
Forest plot depicting subgroup analysis of common mental disorder by screening tools

In this systematic review and meta-analysis, we performed a subgroup analysis based on screening tools in which primary studies used to measure CMD. Among a total 13 primary studies included in this review, seven used the Kessler-10 (K-10) [23, 30–35], two used the HADS [36, 37] and other four used the SRQ-20 [22, 29, 38, 39]. There was a significant difference of CMD reports observed among studies with respect to their screening tools used to measure CMD. The highest (35.20%) and the lowest (17.76%) prevalence of common mental disorder were observed from studies that used the K-10 and the HADS, respectively (Fig. 3).

### Predictors of common mental disorder

From a total of thirteen studies included in this systematic review, only four papers [23, 32, 37, 39] have necessary data to perform meta-analysis for associated factors. The result of the meta-analysis showed that single marital status, HIV/AIDS-related stigma and current job unavailability had statistically significant association with common mental disorder among people with HIV/AIDS.

**Fig. 4:**
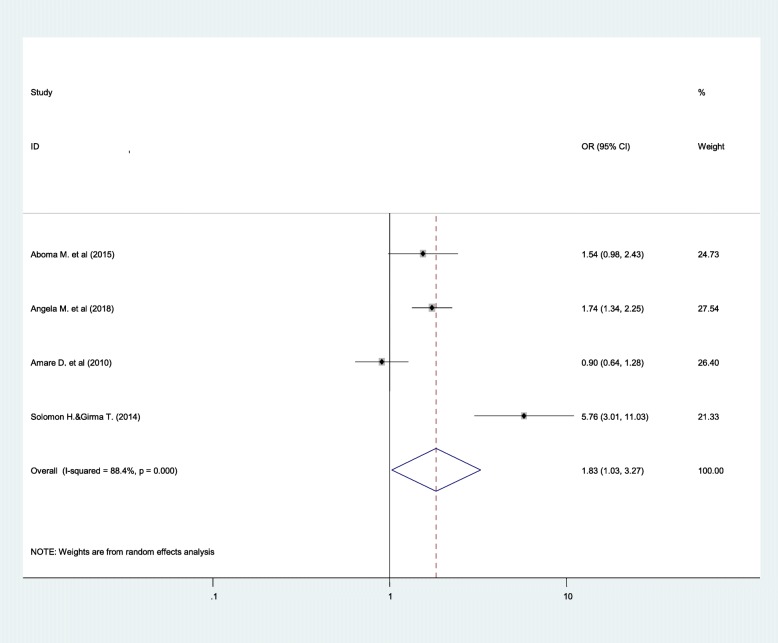
Forest plots depicting pooled odds ratio of marital status with common mental disorder

Accordingly, single individuals were 1.83 times more likely to have common mental disorder as compared to individuals who were married and living together (OR = 1.83; 95% CI: 1.03, 3.27) (Fig. 4).

**Fig. 5:**
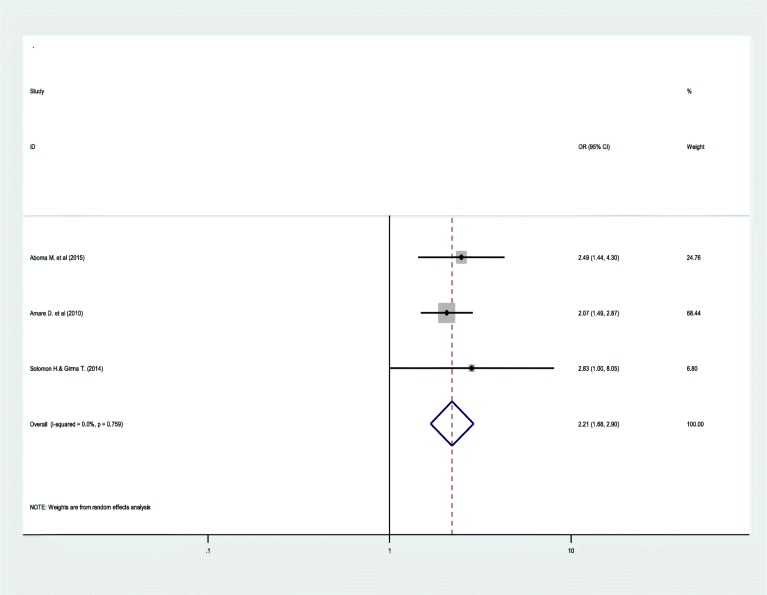
Forest plots depicting pooled odds ratio of stigma with common mental disorder

AIDS-related stigma increases the odds of common mental disorder by 2.21 times among people with HIV/AIDS as compared to individuals without AIDS related stigma (OR = 2.21; 95%CI:1.68, 2.90) (Fig. 5).

**Fig. 6:**
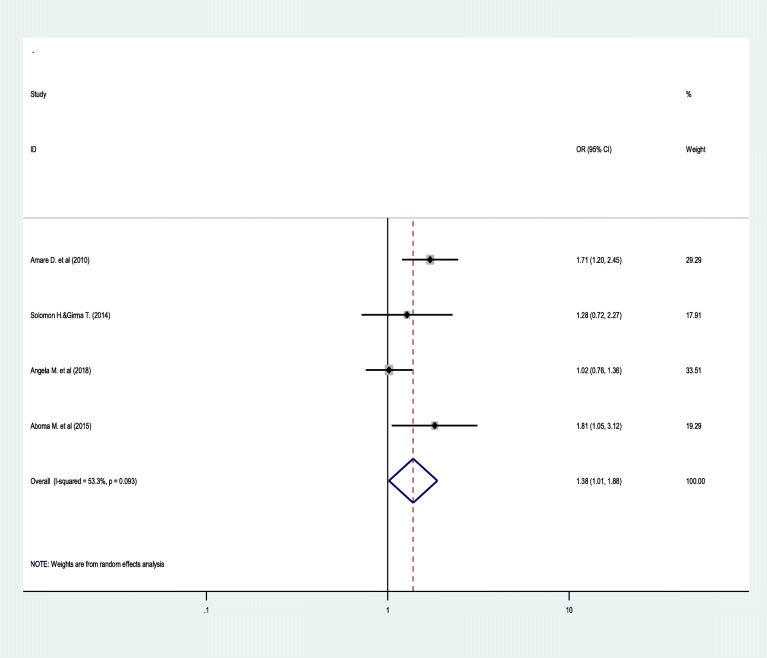
Forest plots depicting pooled odds ratio of job availability with common mental disorder

Similarly, the odds of common mental disorder among people with HIV/AIDS who had no job was increased by 1.38 times as compared to those who were working at their job (OR = 1.38; 95% CI: 1.01, 1.88) (Fig. 6).

## Discussion

Literatures revealed that the prevalence of common mental disorder among people with HIV/AIDS is higher than the general population [40]. The presence of such co-morbid psychiatric disorders can have negative complications by affecting the treatment adherence [15], viral load suppression, immunity [16] and functionality [17] of people with HIV/AIDS. There are some separate studies conducted regarding the magnitude and associated factors of common mental disorder among people with HIV/AIDS in Ethiopia. However, a great variation and inconsistency of reported results are observed across these primary studies. For example, the highest (75%) and the lowest(7.80%) prevalence of common mental disorder were reported from Oromia [23] and Amhara region [22], respectively.

This systematic review showed that the pooled prevalence of common mental disorder among people with HIV/AIDS in Ethiopia was 28.83% with corresponding 95% CI of (17.93, 39.73). The magnitude of common mental disorder in this systematic review and meta-analysis is higher than the reports of global prevalence (17.6%) and WHO estimation in African regions (8%) [8, 9]. The higher magnitude of CMD among people with HIV/AIDS might be explained by the direct brain effects of the HIV virus, presence of opportunistic infections, medication side effects, concomitant psychosocial stressors, and HIV/AIDS-related stigma that might contribute for the psychological and emotional disturbances of people with HIV/AIDS [31].

We also performed a subgroup analysis based on screening tools that primary studies used to screen CMD among PWHA. Accordingly, the result of the sub group analysis showed that there was significant difference on the report of CMD with respect to differences in screening tools of papers. The highest prevalence (35.20%) of CMD was observed among primary articles in which K-10 was used to screen CMD and the lowest prevalence (17.76%) was reported from studies that used HADS. The possible explanation for this variation might be due to the extra ability of the K-10 (unlike HADS and SRQ) to assess somatic like symptoms in addition to depressive and anxiety symptoms [31, 41]. This calls professionals to use a contextualized and standardized screening tool of CMD, particularly applicable for people with HIV/AIDS in Ethiopia.

In this systematic review, predictors of common mental disorder were also identified among people with HIV/AIDS using meta-analysis. The meta-analysis result showed that single marital status, HIV/AIDS-related stigma and unavailability of job had statistically significant association with CMD of people with HIV/AIDS infection.

Accordingly, single individuals were 1.83 times more likely to have common mental disorder as compared to individuals who were married and living together. The possible reason for this significant association can be explained by the fact that people often feel comfortable while they have somebody close to them (particularly, their spouse) to share their psychosocial distress [42]. Moreover, living together with a spouse by itself increases the self-confidence and illness perception that can yield better treatment adherence and improved quality of life of people with HIV/AIDS [43].

The odds of common mental disorder among people with HIV/AIDS-related stigma were increased by 2.21 times as compared to their counterparts. This might be explained by the fact that people with perceived HIV/AIDS-related stigma are more likely to have lower self-image, feeling of inferiority, guilt felling, worthlessness and social isolation which are hallmark symptoms of CMD [14]. In addition, people with HIV/AIDS-related perceived stigma are not often treatment adherent, and may miss their regular/frequent clinic visit. This can lead them to have further psycho-social crisis which predisposes/precipitates symptoms of common mental disorder [44, 45].

Finally, the meta-analysis result showed that the odds of having common mental disorder among people who had no job were 1.38 times higher than those who were working at their job. This might be explained by the fact that people who engage at work can have a better source of income that improves their quality of life, and enable them to develop self-reliance (independency) [46]. Moreover, there is better opportunity for community re-engagement that reduces the HIV/AIDS-related stigma when people start work [47].

## Conclusions

This review showed that the pooled prevalence of common mental disorder among people with HIV/AIDS was higher than the figure reported for the general population. The highest prevalence of common mental disorder was observed among studies used Kessler-10 to screen common mental disorder. Single marital status, HIV/AIDS-related stigma and current job unavailability were identified as factors significantly associated with common mental disorder. This demonstrates a need to integrate mental health and psycho-social support services into the HIV/AIDS care. Prevention of HIV/AIDS-related stigma and accessing job opportunities for people with HIV/AIDS are also highly recommended.

## Supplementary information

**Additional file 1.** PRISMA-P (Preferred Reporting Items for Systematic review and Meta-Analysis Protocols) 2015 checklist: recommended items to address in a systematic review protocol*.

**Additional file 2.** Sample data extraction format.

## Data Availability

All data generated or analysed during this study are included in this article.

## Abbreviations

AIDS: Acquired Immune Deficiency Syndrome
ART: Anti-Retroviral Therapy
CMD: Common Mental Disorder
HAD: Hospital Anxiety and Depression Scale
HIV: Humane Immune Virus
K-10: Kessler-10
SNNPR-: Southern Nation Nationalities and Peoples of Region
SRQ: Self-Reporting Questionnaire
WHO: World Health Organization
YLD-: Years Lived with Disability

## References

[1] Organization WH (2017). Depression and other common mental disorders: global health estimates. World Health Organization.

[2] Mekuriaw B, Zegeye A (2020). Prevalence of Common Mental Disorder and Its Association with Khat Chewing among Ethiopian College Students: A Systematic Review and Meta-Analysis. Psychiatry J.

[3] Lazarus R, Freeman M. Primary-level mental health care for common mental disorder in resource-poor settings: models & practice: A literature review Pretoria: Medical Research Council; 2009. http://svri.org.dedi6.cpt3.host-h.net/sites/default/files/attachments/2016-01-19/primaryhealth.pdf.

[4] Patel V, Araya R, Chowdhary N, King M, Kirkwood B, Nayak S, Simon G, Weiss H (2008). Detecting common mental disorders in primary care in India: a comparison of five screening questionnaires. Psychol Med.

[5] Belayneh Z, Alemu W, Mekuriaw B, Abebe Z (2019). Bipolar spectrum disorders and associated factors among adults attending an antiretroviral therapy clinic in gedeo zone health centers, southern Ethiopia. Neuropsychiatr Dis Treat.

[6] Association AP (2013). Diagnostic and statistical manual of mental disorders (DSM-5®): American psychiatric pub.

[7] Merikangas KR, Kalaydjian A (2007). Magnitude and impact of comorbidity of mental disorders from epidemiologic surveys. Curr Opin Psychiatry.

[8] Steel Z, Marnane C, Iranpour C, Chey T, Jackson JW, Patel V, Silove D (2014). The global prevalence of common mental disorders: a systematic review and meta-analysis 1980–2013. Int J Epidemiol.

[9] Depression W (2017). Other common mental disorders: global health estimates.

[10] Whiteford HA, Degenhardt L, Rehm J, Baxter AJ, Ferrari AJ, Erskine HE, Charlson FJ, Norman RE, Flaxman AD, Johns N (2013). Global burden of disease attributable to mental and substance use disorders: findings from the global burden of disease study 2010. Lancet.

[11] Chibanda D, Mesu P, Kajawu L, Cowan F, Araya R, Abas MA (2011). Problem-solving therapy for depression and common mental disorders in Zimbabwe: piloting a task-shifting primary mental health care intervention in a population with a high prevalence of people living with HIV. BMC Public Health.

[12] Nixon SA, Hanass-Hancock J, Whiteside A, Barnett T (2011). The increasing chronicity of HIV in sub-Saharan Africa: re-thinking" HIV as a long-wave event" in the era of widespread access to ART. Glob Health.

[13] Corrigan PW, River LP, Lundin RK, Wasowski KU, Campion J, Mathisen J, Goldstein H, Bergman M, Gagnon C, Kubiak MA (2000). Stigmatizing attributions about mental illness. J Commun Psychol.

[14] Vanable PA, Carey MP, Blair DC, Littlewood RA (2006). Impact of HIV-related stigma on health behaviors and psychological adjustment among HIV-positive men and women. AIDS Behav.

[15] Nel A, Kagee A (2011). Common mental health problems and antiretroviral therapy adherence. AIDS Care.

[16] Basavaraj K, Navya M, Rashmi R (2010). Quality of life in HIV/AIDS. Indian J Sex Transm Dis.

[17] Pence BW (2009). The impact of mental health and traumatic life experiences on antiretroviral treatment outcomes for people living with HIV/AIDS. J Antimicrob Chemother.

[18] Prentiss D, Power R, Balmas G, Tzuang G, Israelski DM (2004). Patterns of marijuana use among patients with HIV/AIDS followed in a public health care setting. J Acquir Immune Defic Syndr.

[19] Galvan FH, Bing EG, Fleishman JA, London AS, Caetano R, Burnam MA, Longshore D, Morton SC, Orlando M, Shapiro M (2002). The prevalence of alcohol consumption and heavy drinking among people with HIV in the United States: results from the HIV cost and services utilization study. J Stud Alcohol.

[20] Boyer EW, Babu KM, Adkins JE, McCurdy CR, Halpern JH (2008). Self-treatment of opioid withdrawal using kratom (Mitragynia speciosa korth). Addiction.

[21] Mekuriaw B, Belayneh Z, Shemelise T, Hussen R (2019). Alcohol use and associated factors among women attending antenatal care in southern Ethiopia: a facility based cross sectional study. BMC Res Notes.

[22] Basha EA, Derseh BT, Haile YGE, Tafere G (2019). factors affecting psychological distress among people living with HIV/AIDS at selected hospitals of north Shewa zone, Amhara region, Ethiopia. AIDS Res Treat.

[23] Parcesepe AM, Tymejczyk O, Remien R, Gadisa T, Kulkarni SG, Hoffman S, Melaku Z, Elul B, Nash D (2018). Household decision-making power and the mental health and well-being of women initiating antiretroviral treatment in Oromia, Ethiopia. AIDS Care.

[24] Liberati A, Altman DG, Tetzlaff J, Mulrow C, Gøtzsche PC, Ioannidis JP, Clarke M, Devereaux PJ, Kleijnen J, Moher D (2009). The PRISMA statement for reporting systematic reviews and meta-analyses of studies that evaluate health care interventions: explanation and elaboration. PLoS Med.

[25] Peterson J, Welch V, Losos M, Tugwell P (2011). The Newcastle-Ottawa scale (NOS) for assessing the quality of nonrandomised studies in meta-analyses.

[26] Rücker G, Schwarzer G, Carpenter JR, Schumacher M (2008). Undue reliance on I 2 in assessing heterogeneity may mislead. BMC Med Res Methodol.

[27] Sterne JA, Egger M (2001). Funnel plots for detecting bias in meta-analysis: guidelines on choice of axis. J Clin Epidemiol.

[28] Egger M, Smith GD, Schneider M, Minder C (1997). Bias in meta-analysis detected by a simple, graphical test. Bmj.

[29] Selamawit Z, Nurilign A. Common mental disorder among HIV infected individuals at Comprehensive HIV Care and Treatment Clinic of Debre Markos referral Hospital, Ethiopia. J AIDS Clin Res. 2015;6(2):420. 10.4172/2155-6113.1000420.

[30] Mousley E, Deribe K, Tamiru A, Tomczyk S, Hanlon C, Davey G. Mental distress and podoconiosis in northern Ethiopia: a comparative cross-sectional study. Int Health. 2015;7(1):16–25. 10.1093/inthealth/ihu043 PMC4236095 25062906

[31] Deribew A, Deribe K, Reda AA, Tesfaye M, Hailmichael Y, Maja T (2013). Do common mental disorders decline over time in TB/HIV co-infected and HIV patients without TB who are on antiretroviral treatment?. BMC Psychiatry.

[32] Deribew A, Tesfaye M, Hailmichael Y, Apers L, Abebe G, Duchateau L, Colebunders R (2010). Common mental disorders in TB/HIV co-infected patients in Ethiopia. BMC Infect Dis.

[33] Soboka M, Tesfaye M, Feyissa GT, Hanlon C (2014). Alcohol use disorders and associated factors among people living with HIV who are attending services in south West Ethiopia. BMC Res Notes.

[34] Soboka M, Tesfaye M, Feyissa GT, Hanlon C (2015). Khat use in people living with HIV: a facility-based cross-sectional survey from south West Ethiopia. BMC Psychiatry.

[35] Parcesepe AM, Tymejczyk O, Remien R, Gadisa T, Kulkarni SG, Hoffman S, Melaku Z, Elul B, Nash D (2018). Psychological distress, health and treatment-related factors among individuals initiating ART in Oromia, Ethiopia. AIDS Care.

[36] Tesfaw G, Ayano G, Awoke T, Assefa D, Birhanu Z, Miheretie G, Abebe G (2016). Prevalence and correlates of depression and anxiety among patients with HIV on-follow up at alert hospital, Addis Ababa, Ethiopia. BMC Psychiatry.

[37] Tesfaye SH, Bune GT (2014). Generalized psychological distress among HIV-infected patients enrolled in antiretroviral treatment in Dilla University hospital, Gedeo zone, Ethiopia. Glob Health Action.

[38] Duko B, Toma A, Abraham Y (2019). Prevalence and correlates of common mental disorder among HIV patients attending antiretroviral therapy clinics in Hawassa City, Ethiopia. Ann General Psychiatry.

[39] Motumma A, Negesa L, Hunduma G, Abdeta T (2019). Prevalence and associated factors of common mental disorders among adult patients attending HIV follow up service in Harar town, eastern Ethiopia: a cross-sectional study. BMC Psychology.

[40] Myer L, Smit J, Roux LL, Parker S, Stein DJ, Seedat S (2008). Common mental disorders among HIV-infected individuals in South Africa: prevalence, predictors, and validation of brief psychiatric rating scales. AIDS Patient Care STDs.

[41] Reda AA (2011). Reliability and validity of the Ethiopian version of the hospital anxiety and depression scale (HADS) in HIV infected patients. PLoS One.

[42] Kiecolt-Glaser JK, Newton TL (2001). Marriage and health: his and hers. Psychol Bull.

[43] Gonzalez JS, Penedo FJ, Antoni MH, Durán RE, McPherson-Baker S, Ironson G, Isabel Fernandez M, Klimas NG, Fletcher MA, Schneiderman N (2004). Social support, positive states of mind, and HIV treatment adherence in men and women living with HIV/AIDS. Health Psychol.

[44] Reilly KH, Clark RA, Schmidt N, Benight CC, Kissinger P (2009). The effect of post-traumatic stress disorder on HIV disease progression following hurricane Katrina. AIDS Care.

[45] Mekuriaw B, Belayneh Z, Yitayih Y (2020). Magnitude of Khat use and associated factors among women attending antenatal care in Gedeo zone health centers, southern Ethiopia: a facility based cross sectional study. BMC Public Health.

[46] Schrimshaw EW (2002). Social support, conflict, and integration among women living with HIV/AIDS 1. J Appl Soc Psychol.

[47] Whetten K, Reif S, Whetten R, Murphy-McMillan LK (2008). Trauma, mental health, distrust, and stigma among HIV-positive persons: implications for effective care. Psychosom Med.

