# Correction to: Magnitude and predictors of common mental disorder among people with HIV/AIDS in Ethiopia: a systematic review and meta-analysis

**DOI:** 10.1186/s12889-020-09017-5

**Published:** 2020-06-08

**Authors:** Zelalem Belayneh, Birhanie Mekuriaw, Tsegaye Mehare, Seid Shumye, Mekonnen Tsehay

**Affiliations:** 1 Department of Psychiatry, College of Health and Medical Science, Dilla University, Dilla, Ethiopia; 2 Bio-Medical Department, College of Health and Medical Science, Dilla University, Dilla, Ethiopia; 3 Department of Psychiatry, College of Medicine and Health Science, Wollo University, Dessie, Ethiopia

**Correction to: BMC Public Health (2020) 20:689**

**https://doi.org/10.1186/s12889-020-08800-8**

It was highlighted that the original article [1] contained an error in the name of Seid Shumye. This was incorrectly captured as Saide Shumy. This Correction article clarifies the correct spelling of the author’s first and last name. The original article has been updated.
